# Rare Escherichia coli Empyema Necessitating to Pelvic Retroperitoneum With Extension to the Groin

**DOI:** 10.7759/cureus.47467

**Published:** 2023-10-22

**Authors:** Sharmeen Azad, Andrew McCague, Austin Henken-Siefken, Tracy Taggart

**Affiliations:** 1 Surgery, Desert Regional Medical Center, Palm Springs, USA; 2 Medicine, Western University of Health Sciences, Lebanon, USA; 3 Trauma, Desert Regional Medical Center, Palm Springs, USA

**Keywords:** necessitating to retroperitoneum, necessitating, e. coli, e.coli empyema, retroperitoneum abscess, empyema necessitasis

## Abstract

Empyema necessitans is a rare form of infection that spreads to the soft tissue of visceral organs. In the case of thoracic empyema, the infection can spread to the parietal pleura of the lungs. This can cause many complications as treatment is more complicated with the spread of this infection. *Escherichia coli (E. coli)* is a rare occurrence of this infection, and understanding its role in the community and the implications of its survival in extraintestinal environments can be beneficial for the treatment of these cases.

## Introduction

Empyema is a collection of pus in the pleural space. A rare condition known as empyema necessitans (EN) is described as a sneaky spread of the empyema via the parietal pleura and dissection into the subcutaneous tissue [1]. The chest wall, peritoneum, pericardium, retroperitoneum, esophagus, mediastinum, abdominal wall, paravertebral space, vertebrae, bronchus, breast, and diaphragm have all been identified as places where empyema might spread [2]. 

We describe a 60-year-old female with diffuse abdominal pain whose laboratory values indicated sepsis. CT scans of the abdomen, pelvis, and chest confirmed severe infection that extended from the base of the chest into the left inguinal region. A thoracostomy tube was placed for fluid drainage, and the patient was started on piperacillin-tazobactam in the emergency department. We present this case of empyema necessitating the groin and its diagnostic difficulties. 

## Case presentation

**Figure 1:**
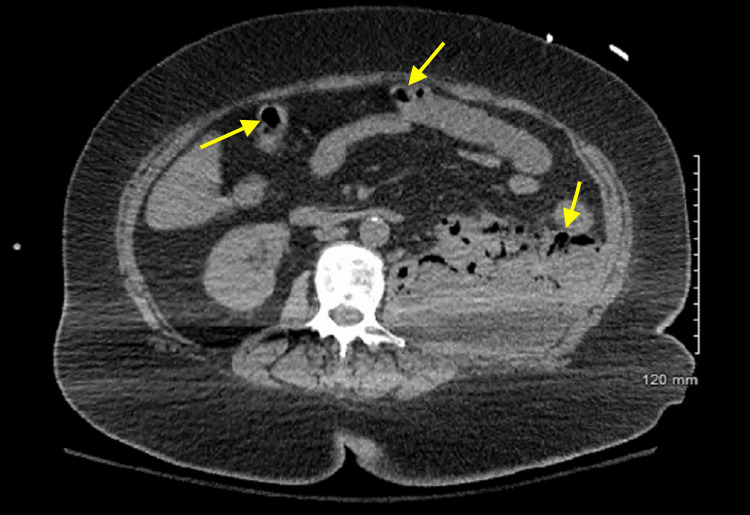
Computed tomography of the chest demonstrating retroperitoneal air within various parts of the colon (yellow arrows), which is concerning for necrotizing fasciitis

The patient is a 60-year-old female with a past medical history of diabetes, hypertension, and hyperlipidemia who was transferred to our center from an outside facility for a higher level of care. The patient was transferred for possible necrotizing fasciitis of the left retroperitoneum. The patient was confused, agitated, and writhing in pain on arrival. The patient's chief complaint was diffuse abdominal pain. In the ED, the patient was hypotensive, which improved with IV fluids. Laboratory results showed her initial WBC at 25.8k/mm3 (normal range 4-11 k/mm3) upon arrival, indicating sepsis. A CT scan of the abdomen and pelvis done at an outside facility shows extensive abnormal soft tissue thickening and gas throughout the left retroperitoneum, extending from the visualized base of the chest into the left inguinal region and proximal thigh. These findings were concerning for extensive infectious processes with phlegmon and necrotizing fasciitis. There was partial visualization of gas in the anterior lower left chest, which was concerning for pneumothorax. A CT scan of the chest reveals left-sided pneumothorax, less than 10%, with a large left-sided pleural effusion (Figure 1). A thoracostomy tube was placed in the ED, which drained roughly 700ml of purulent fluid. The patient was started on piperacillin-tazobactam at this time.

The patient was taken to the operative room (OR) emergently for an exploratory laparotomy with drainage of the visualized left retroperitoneal abscess. During the procedure, no perforations or other abnormalities were found. The abscess was drained, cultured, and washed. The abdomen was then left open with an AbThera, which provides an active temporary abdominal closure system in place. The patient was taken back to the OR the following day, where another exploratory laparotomy and washout were done. At this time, it was noted that the abscess extended down into the pelvis to the distal rectum, all retroperitoneal, so due to concern of rectal perforation and occult rectal pathology, it was decided to proceed with a sigmoidectomy with end colostomy and wide drainage. The fascia was closed at this time. Earlier cultures of the abscess resulted in infection with *E. coli*, sensitive to piperacillin-tazobactam. Previously ordered piperacillin-tazobactam was continued, and vancomycin was started at this time. The patient was again taken back to the OR on admission day three, where a left video-assisted thoracic exploration was done. Purulent fluid was noted throughout, and a fibrinous peel was visualized over the pleural surface. Repeat cultures were taken, confirming *E. coli* infection. The thoracic cavity was irrigated, and three chest tubes were placed. The patient was then transferred to ICU post-op. 

Over the course of admission, the WBC count rapidly declined from 25.8k/µl to 10.0k/µl. Extubation was done on admission day five. Chest tube output continued to decline and was later removed. The patient was discharged in good condition. 

## Discussion

Empyema necessitans (EN) is a rather uncommon condition in which the empyema slowly extends into the parietal pleura and then dissects into the subcutaneous tissue of the chest wall [1]. The most common causes of empyema include S*taphylococcus aureus *and *Pseudomonas aeruginosa;* however, in rare cases, such as the one described by Sabir et al. and Crouch et al. [3,4], empyema can be caused by *E. coli*.

This is more common in patients with comorbidities such as cirrhosis or immunocompromised patients, as described by Jang et al. and Tao et al. [5,6]. In that case, *E. coli *empyema was found to be more prominent in patients who have comorbidities as compared to patients who did not. This is most likely because elderly patients with co-occurring chronic conditions, such as diabetes mellitus, renal disease, and alcoholism, are more susceptible to infections than the general population [3]. Depending on the patient's risk factors, empyema is linked to a high mortality incidence of 5.4% to 22% [7]. The high mortality rate of this condition makes it a very important topic of discussion. Recent research has shown that some specific *E. coli *strains can persist for extended periods of time and even have the potential to reproduce in extra-intestinal settings [8].

There have been various cases that have been presented with empyema necessitans [1-10]. Pneumonia, thoracic trauma, iatrogenic treatments such as thoracentesis and heart surgery, bronchopleural fistula, esophageal rupture, and extension of infection from the abdomen and mediastinum are just a few causes of thoracic empyema that are frequently cited [3]. On the other hand, non-parapneumonic empyema differs depending on the underlying source of infection and can be brought on by a wide range of pathogens. Eight to ten percent of cases are caused by *Escherichia coli (E. coli)* and other gram-negative Enterobacteriaceae [3].

According to Banderu et al. [1], piperacillin-tazobactam and vancomycin are the best antibiotic treatments for a case of thoracic empyema caused by *E. coli* that necessitates any area of the body. Plain radiographs are frequently nonspecific and occasionally even normal in the diagnosis of EN. Plain radiographs may, at most, indicate a density of soft tissues in the chest wall. The best way to determine the severity of an infection outside the thoracic cavity is with a chest CT scan. Other EN treatment options include pleural space drainage, either open or closed, to enlarge the lung and reduce fibrosis risks [1]. 

In our case, we had a 60-year-old female patient who came in with findings that were concerning for extensive infectious processes with phlegmon and necrotizing fasciitis. Lab cultures of the abscess showed infection with *E. coli*. This was particularly unusual as *E. coli* is a very rare cause of pneumonia. *E. coli* can infect tissues or organs that are susceptible to the virulence factors present in colonizing strains outside of the gastrointestinal tract [7]. The empyema might have been caused by an intestinal condition if normal intestinal flora were seen [8]. This led us to question whether our patient had some source of gastrointestinal infection or malignancy. Though there is some research on thoracic empyema, the topic of diagnostic difficulties has not been explored before. This case report attempted to underline these difficulties. Some differential diagnoses that were considered for this patient, given her clinical presentation, were bowel perforation, appendicitis, and cholecystitis. However, after extensive imaging and lab work, there was no indication of any of these differentials.

Our case showcases a unique aspect of thoracic empyema. Our patient's empyema spread from her thoracic region down to the pelvis. In this presentation, the most important question to address was where the source of infection was. Our initial thoughts pointed towards an abdominal origin, which is when we performed a sigmoidectomy as a precaution. However, after a full set of exploration, we found the infection to be in the chest and the groin. We believe this case is important in underlining the need to act urgently in picking up on rare bacterial infections that can lead to death very quickly. 

A limiting factor to consider for this case is that this is a single case. It is important to acknowledge that outcomes may be different with other patients, but based on the information we had available, this was our observation and treatment for the patient. Additionally, there was no definitive proof that the infection originated in the thorax, but we made this diagnosis by ruling out all other potential spaces of origin. 

## Conclusions

Our patient presented with diffuse abdominal pain and was identified to be in septic shock at the time of presentation. After a thorough work-up and multiple abdominal surgeries, we found *E. coli* to be the causative agent of the infection. The origin of the infection is presumed to be caused by* E. coli*, which was possibly caused by the extraintestinal manifestation of *E. coli*. Our case showed that there can be rare causes of extraintestinal *E. coli* infections and that investigating the role of *E. coli* in the community can be very beneficial for future cases. 
